# TrueNTH sexual recovery study protocol: a multi-institutional collaborative approach to developing and testing a web-based intervention for couples coping with the side-effects of prostate cancer treatment in a randomized controlled trial

**DOI:** 10.1186/s12885-017-3652-3

**Published:** 2017-10-02

**Authors:** D. Wittmann, A. Mehta, L. Northouse, R. Dunn, T. Braun, A. Duby, L. An, L. Arab, R. Bangs, S. Bober, J. Brandon, M. Coward, M. Dunn, M. Galbraith, M. Garcia, J. Giblin, M. Glode, B. Koontz, A. Lowe, S. Mitchell, J. Mulhall, C. Nelson, K. Paich, C. Saigal, T. Skolarus, J. Stanford, T. Walsh, C. E. Pollack

**Affiliations:** 1 University of Michigan, 2800 Plymouth Road, Bldg. 16, Rm 110E, Ann Arbor, MI 48109-2800 USA; 2 Emory University, Atlanta, GA USA; 3 University of California-Los Angeles, California, Los Angeles USA; 4 Dana Farber Cancer Center and Harvard University, Boston, MA USA; 5 University of North Carolina, Chapel Hill, NC USA; 6 University of Colorado-Denver, Denver, CO USA; 7 University of California-San Francisco, San Francisco, CA USA; 8 Duke University, Durham, NC USA; 9 Prostate Cancer Foundation-Australia, St Leonards, Australia; 10 Memorial Sloan Kettering Cancer Center, New York City, NY USA; 11 TrueNTH Movember Foundation, Michigan, USA; 12 Fred Hutchinson Comprehensive Cancer Center, Seattle, Washington USA; 13 University of Washington, Seattle, Washington USA; 14 Johns Hopkins University, Baltimore, MD USA; 15 VA Ann Arbor Healthcare System, HSRD Center for Clinical Management Research, Ann Arbor, USA

**Keywords:** Prostate cancer sexual recovery cancer survivorship intervention

## Abstract

**Background:**

Over half of men who receive treatment for prostate suffer from a range of sexual problems that affect negatively their sexual health, sexual intimacy with their partners and their quality of life. In clinical practice, however, care for the sexual side effects of treatment is often suboptimal or unavailable. The goal of the current study is to test a web-based intervention to support the recovery of sexual intimacy of prostate cancer survivors and their partners after treatment.

**Methods:**

The study team developed an interactive, web-based intervention, tailored to type of treatment received, relationship status (partnered/non-partnered) and sexual orientation. It consists of 10 modules, six follow the trajectory of the illness and four are theme based. They address sexual side effects, rehabilitation, psychological impacts and coaching for self-efficacy.

Each includes a video to engage participants, psychoeducation and activities completed by participants on the web. Tailored strategies for identified concerns are sent by email after each module. Six of these modules will be tested in a randomized controlled trial and compared to usual care. Men with localized prostate cancer with partners will be recruited from five academic medical centers. These couples (*N* = 140) will be assessed prior to treatment, then 3 months and 6 months after treatment. The primary outcome will be the survivors’ and partners’ Global Satisfaction with Sex Life, assessed by a Patient Reported Outcome Measure Information Systems (PROMIS) measure. Secondary outcomes will include interest in sex, sexual activity, use of sexual aids, dyadic coping, knowledge about sexual recovery, grief about the loss of sexual function, and quality of life. The impact of the intervention on the couple will be assessed using the Actor-Partner Interaction Model, a mixed-effects linear regression model able to estimate both the association of partner characteristics with partner and patient outcomes and the association of patient characteristics with both outcomes.

**Discussion:**

The web-based tool represents a novel approach to addressing the sexual health needs of prostate cancer survivors and their partners that—if found efficacious—will improve access to much needed specialty care in prostate cancer survivorship.

**Trial registration:**

Clinicaltrials.gov registration # NCT02702453, registered on March 3, 2016.

## Background

Research on older adults has found that couples who retain their sexual relationships have a better quality of life (QOL) [1–4] whereas disruption of these relationships can lead to distancing, loneliness, and depression [1, 5]. Being in a relationship is associated with improved cancer survival and sexual relationships are an important source of resilience for patients with cancer [2]. Among men with prostate cancer, treatment-related erectile dysfunction (ED) [3] has a detrimental impact on survivors’ lives, on their partners and on their relationships [4, 6]. Although it is important for men and their partners to retain sexual viability after prostate cancer treatment, there are currently no guidelines for helping men and couples manage post-treatment sexual health. As a result, couples seldom receive the help they need in this aspect of prostate cancer survivorship.

While sexuality has biopsychosocial components, current management of the sexual side effects of prostate cancer treatment focuses only on treating treatment-related physiologic dysfunction – ED - with various erectile aids [7]. Studies indicate that many men who stand to benefit from these treatments do not utilize them if the psychosocial aspects of sexuality are not addressed [8–10]. The psychosocial aspects of sexuality include feelings about one’s sexual function, ability to feel pleasure, confidence as a lover and skill with which to navigate a sexual relationship [11]. Some psychosocial factors can become potentially influential barriers to sexual recovery after prostate cancer treatment, such as unrealistic expectations of functional recovery, lack of knowledge about sexual rehabilitation, unresolved grief about sexual losses, couple conflict, and extensive family responsibilities [12, 13]. Studies have shown that this biopsychosocial approach to post-prostate cancer sexual recovery can be effective [14].

Patients and providers frequently lack an understanding of the complexity of sexuality, and are uncomfortable with the subject [15–17]. Providers are generally unprepared for these conversations and are unaware of sexual health resources. At times, they are uncertain about some available therapies. For example, although there is increasing evidence supporting the safety of testosterone replacement therapy (TRT) in some prostate cancer survivors, many providers are unsure about when to initiate therapy, or how to appropriately monitor patients on TRT [18]. Access to sexual health expertise that focuses not only on physiologic ED treatments but also on the psychosocial aspects of sexuality [11] is frequently unavailable. Many insurances do not cover medications and devices as well as sexual dysfunction diagnoses despite the fact that those diagnoses are a part of the mental health insurance codes and their coverage could facilitate access to sexual health treatment [19]. As a result, the healthcare system does not adequately support patients and partners in their pursuit of sexual health after prostate cancer treatment.

Recognizing the multiple, overlapping barriers to sexual recovery after prostate cancer treatment, researchers are designing interventions specifically aimed at improving sexual outcomes. Although small in number, interventions that have attempted to address the psychosocial aspects of sexuality have shown a number of positive outcomes such as stress reduction, temporary improvement in sexual function, increased knowledge about rehabilitation, more realistic expectations about the recovery of sexual function, use of pro-erectile aids and higher levels of relationship satisfaction [14, 20–27]. Studies have also shown that physical exercise can have a positive effect on the recovery of erectile function, and on the maintenance of a masculine self-image [28, 29]. Evidence continues to be mixed and the search for meaningful measurable outcomes is still in process [30].

Beyond the development of suitable interventions for couples, other gaps exist. One such gap is the lack of dissemination of tested interventions in prostate cancer beyond the research setting. Another is the lack of research that incorporates important sociodemographic factors, cultural differences, and sexual orientation that may impact sexual recovery after prostate cancer. There are very few studies about African American men with prostate cancer [31]. There is a paucity of research on the attitudes of men from other racial/ethnic groups that may be used to inform the tailoring of interventions [32]. Similarly, research on gay men with prostate cancer has been largely missing in the literature [33], thus leaving as many as 11,000 gay men with prostate cancer each year without attention to their specific sexual health care needs [34]. There is no research on the effect of prostate cancer-related sexual dysfunction on men who are not partnered. Finally, few studies focus on men treated with androgen deprivation therapy (ADT) as health care providers may mistakenly assume that sexual recovery is not an important consideration for these patients [35, 36]. Preferences based on cultural background, sexual orientation, and an understanding of men on ADT need to be incorporated into sexual health interventions so that the interventions can be tailored to these survivors’ needs.

Web-based interventions may be an important mode of delivery of sexual recovery interventions for prostate cancer survivors. They can address multiple barriers to dissemination and allow for a tailored approach. Although prostate cancer patients and partners are primarily an older cohort, they are frequently internet-literate and have shown a high interest in and ability to collaborate with providers online [37–39]. Web-based interventions may be especially useful for patients who live far away from the clinic, for those for whom clinic visits may pose an economic burden (e.g., those whose travel costs are high or who have difficulty taking time away from work), and for those survivors who find topics such as sexual health difficult to approach in a face-to-face visit. A web-based approach has also been shown to be valuable to cancer patients with fewer social support resources [40]. In prostate cancer, couples, not just individual men with prostate cancer have also benefited from a web-based intervention aimed at reducing distress [41]. In the setting of sexual recovery, an intimacy enhancement and psychoeducational sexual health intervention was shown to have been equally acceptable to patients in web-based and face-to-face formats [20].

In this manuscript, we describe a novel intervention that delivers web-based content for sexual recovery for men with prostate cancer. The intervention is tailored based on partnership status, treatment type and sexual orientation, with sensitivity to cultural differences. It is designed to allow men and their partners to participate in it together. We present the theoretical model which underlies the intervention approach. The study design, assessments, and analytic approach are also presented.

### The national collaborative

The Movember Foundation has collaborated with national non-profit organizations, such as the Livestrong Foundation (United States) and the Prostate Cancer Foundation (Australia, the United Kingdom, the United States and Canada) to offer funding to selected institutions with a clinical and research track record in prostate cancer survivorship. A Request for Proposals in the United States was issued in the fall of 2013, and 15 institutions were selected: Johns Hopkins University, Emory University, Harvard University, Memorial Sloan Kettering Cancer Center, Moffitt Cancer Center, University of California San Francisco, Los Angeles and Davis, University of Michigan, University of Washington, Oregon Health Sciences University, University of Colorado-Denver, Wayne State University, Duke University and University of North Carolina. In March 2014, representatives from the 15 institutions met to define priorities for interventions, and agreed to participate in building those interventions in which they had the most experience. Sexual Recovery was one of the priorities selected for intervention building.

The Sexual Recovery Intervention was developed by a team (i.e., the Full Team) of sexual health experts, urologists, psychologists, health services researchers, epidemiologists, primary care physicians, radiation oncologists, medical oncologists, nurses (one of these professionals is a survivor and another one is a partner), and survivor and partner advocates from 11 institutions (Emory University, University of Colorado-Denver, University of California-Los Angeles, Johns Hopkins University, Fred Hutchinson Cancer Research Center, University of Michigan, Duke University, Memorial Sloan Kettering and Harvard University, University of North Carolina, University of California-San Francisco). A Design Team was established to write a proposal for intervention development and testing. It included a sexual health expert, a primary care physician, a urologist, and an internal medicine physician with expertise in information technology. The Full Team participated in monthly teleconferences and also provided regular electronic editorial contributions. After peer review by the Scientific Committee of the Movember Foundation, the protocol was amended and re-submitted for funding. Funding was assigned in March 2015.

The intervention was developed at the University of Michigan, given the institution’s expertise in sexual health and technology. However, the Design Team and the Full Team provided critical feedback during the development phase. The intervention is currently being tested in a multi-center randomized controlled trial (RCT) involving five institutions in the United States (Emory University, University of California-Los Angeles, Memorial Sloan Kettering Cancer Center, Johns Hopkins University and the University of Michigan). Plans are in progress to adapt the intervention in the United Kingdom.

The intervention, once tested, will be integrated into a support structure for prostate cancer survivors, partners, and families, which will be available on the Movember Foundation website. The prioritized US interventions have a collective over-arching identity as True NTH (pronounced ‘True North’). Its goal is “to improve the physical and mental well-being of men living with prostate cancer, together with their partners, caregivers and families.”

### A theoretical model of sexual recovery after prostate cancer treatment

The proposed web-based sexual recovery intervention is underpinned by a theoretical model that benefits from existing research. It was tested in a mixed methods study to ensure content validity by the principal investigator and colleagues [42]. It is based on the biopsychosocial understanding of sexuality [11, 43]. Recognizing that ED and subsequent changed sexuality represent loss for both the man and the partner [44, 45], the model incorporates concepts from the grief literature to suggest that sexual recovery includes, at least in part, a grief process [46, 47].

### Study overview

The study’s first step was to develop the intervention and conduct formative testing with 3 focus groups which included 4 heterosexual couples, 8 survivors and 8 partners, respectively. Two non-heterosexual couples were interviewed separately as we were unable to recruit for a full focus group. Usability testing was conducted with 5 couples and 5 individual survivors who commented, while working through the intervention, on the ease of use, relevance, content and graphics. The majority of the participants endorsed the web-based format and suggested that the intervention include preparation for the sexual side-effects, rehabilitation, preparation for the emotional impact of erectile dysfunction and diminished libido on couples, education for partners about the importance of erections to men, sensitivity to gay sex and education of providers regarding sexuality of men who have sex with men [48].

**Fig. 1:**
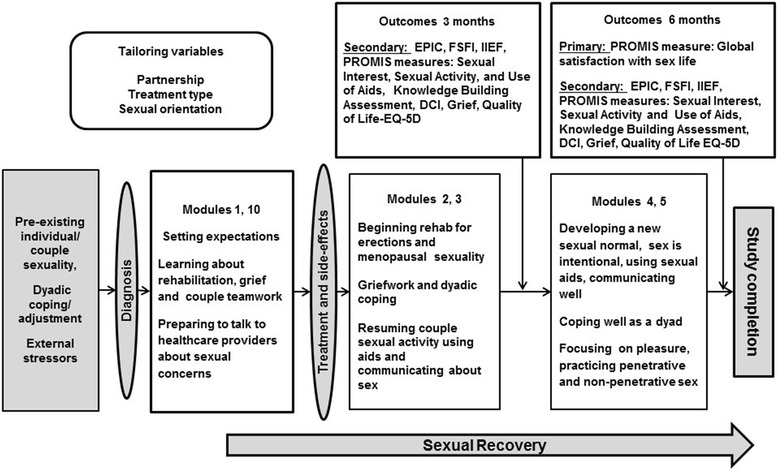
Tailored web-based intervention to support couples’ sexual recovery after treatment for localized prostate cancer (RCT of 6 modules in a pre-treatm6nt to 6-month post-treatment time-frame)

Figure 1 illustrates the study process for the intervention group, starting prior to diagnosis with preparation for the sexual side-effects, their impacts and rehabilitation, and continuing with support for sexual recovery through 6 months after treatment (also the study completion). The figure also illustrates where the intervention modules of the TrueNTH Sexual Recovery Intervention will aim to affect the process of couples’ recovery. One of the modules (M10) coaches patients and partners to engage comfortably in discussions with healthcare providers about sexual problems and solutions. Finally, the figure includes measurement of the effect of the intervention on outcomes proposed in this protocol.

This study aims to evaluate the effect of 6 modules of the intervention in an RCT on patients’ and partners’ satisfaction with their sex life, compared to usual care at 6 month follow up. It is hypothesized that patients and partners who participate in the online intervention will report more satisfaction with their sex life than the control group. The intervention group is also expected to report greater knowledge about sexual recovery, less intense grief about sexual losses, more interest in sex, greater use of sexual aids, better dyadic coping, and a higher quality of life than the control group after treatment.

## Methods

### Study setting

Recruitment will take place in urology and radiation oncology clinics at participating sites in the True NTH consortium: University of Michigan, Emory University, Johns Hopkins University, University of California –Los Angeles and Memorial Sloan Kettering Cancer Center. Sites were selected based on (1) patient volume which will impact feasibility and recruitment costs, (2) diversity of the patient population, and (3) current standard of care regarding sexual health. Each site will have a site PI and a study coordinator. The website’s survey and intervention couple engagement logistics data will be centrally managed at the University of Michigan which will also serve as the coordinating site for clinical data and a provider of technical support for all participating sites. Potential participants will be identified by a member of the study team at participating sites through screening clinic and surgical schedules as well through referral by the treating physician. The goal for each site will be to recruit a minimum of 15 couples and a maximum of 50 couples.

### Participant selection

#### Inclusion criteria

Patients diagnosed with localized prostate cancer will be eligible if they are planning to receive definitive treatment within 3–6 weeks after diagnosis with either radiation or prostatectomy without ADT. Participants must be in a 6 month or longer relationship with a spouse/partner who is willing to sign an informed consent. Informed consent must be signed at least 2 weeks prior to the start of treatment so that the participants will have time to complete baseline surveys and access the first module of the intervention. Additionally, participants must be able to speak or read English without cognitive deficit, not be in acute psychiatric crisis, have reliable internet access with separate addresses for the patient and partner, and be 18 years of age or older.

### Randomization

**Fig. 2:**
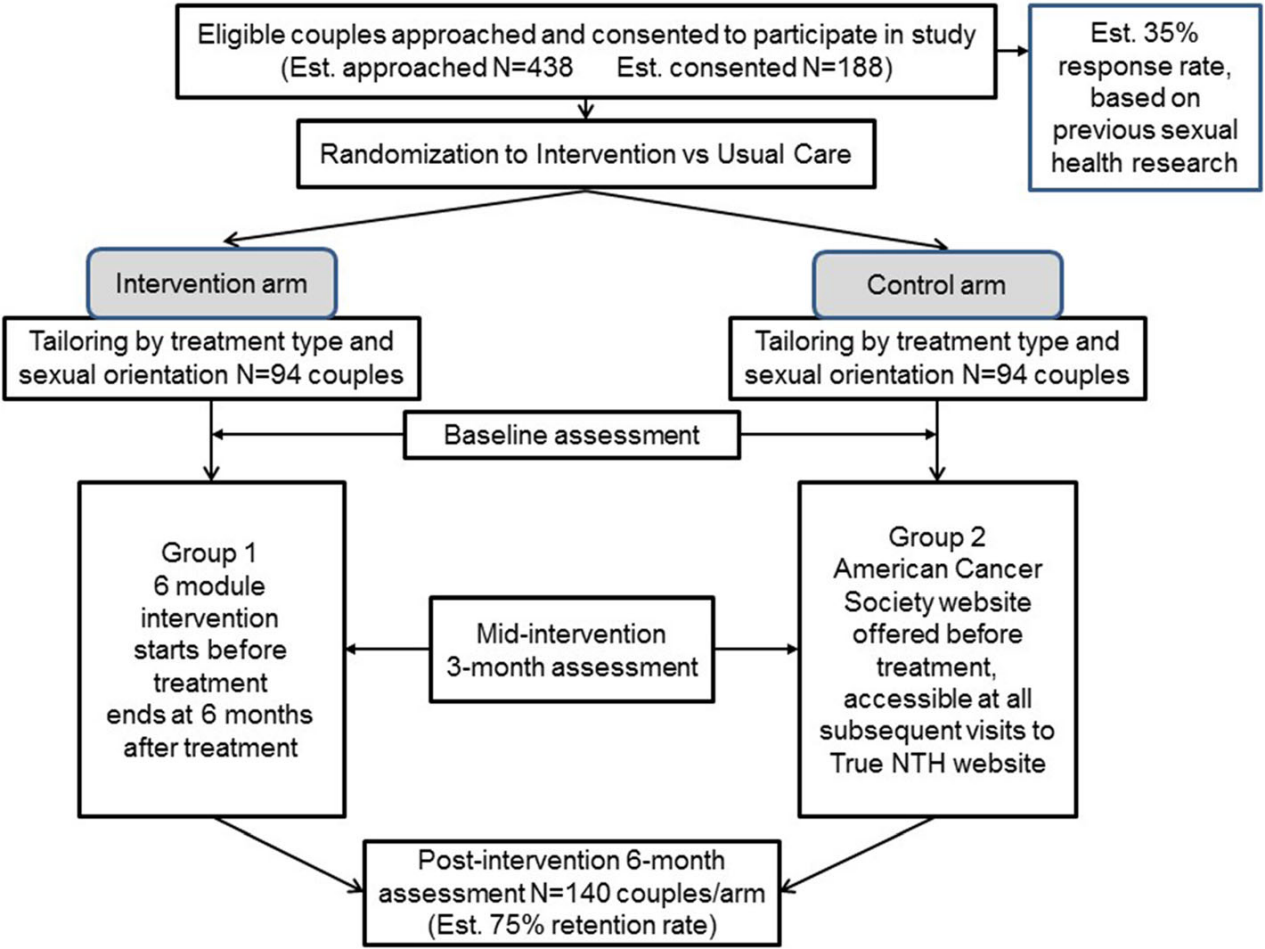
Study progression

The patient/partner dyad will be randomized to either the intervention arm or the usual care arm after completion of the informed consents, and prior to initiation of treatment. Randomization will be performed using a computer-generated randomized block design to minimize the potential for sample size differences between the two arms. The randomized block design will be stratified by enrollment site to facilitate ease of administration of the trial (Fig. 2). Participants randomized to usual care will be routed to the American Cancer Society’s web page that provides information about the sexual side-effects of cancer and sexual rehabilitation, which can be considered a part of usual care. Participants will be blinded as to the arm that they are entering by language at recruitment which will indicate that we are comparing two supportive interventions for sexual recovery after prostate cancer treatment. Participants will be unblinded by letter after they complete their 6-month survey.

### Intervention

The full intervention will consist of 10 web-based modules, 6 of which are consistent with the trajectory of prostate cancer and its treatment (Table 1). Four modules will be thematically linked to the experience of sexual recovery after prostate cancer treatment.

**Table 1:** TrueNTH Sexual Recovery Intervention Content

Module	Theme	Goal	Content
1	Pre-treatment education	Preparation for the sexual side-effects of prostate cancer treatment, rehabilitation, emotional response and working on recovery as a team.	Video: couple modeling acceptance of working together on sexual recovery.
			Education: etiology and nature of treatment-related sexual dysfunction, its effect on relationships, goal and type of rehabilitation, individual and couple emotional work that supports recovery.
			Activity: sharing concerns and goals for recovery.
			Request for tailored strategies: specific concerns will trigger a tailored response delivered to patient and/or partner by email.
2	Preparation for the resumption of sexual activity	Understanding of sexual rehabilitation as it relates to erectile dysfunction, female post-menopausal challenges; normalizing of feelings of grief about sexual losses; maintenance of feeling connected in the face of the sexual challenges ahead.	Video: patient discussing changes after prostate cancer treatment and engagement in rehabilitation
			Education: description of penile rehabilitation methods, maintenance of vaginal health and taking pressure off the sexual relationship during the early phase of rehabilitation.
			Activity: treatment-related sexual changes and post-menopausal changes, determination of use of sexual aids for rehabilitation by both man and partner, introduction of rehabilitation diary, staying connected.
			Request for tailored strategies: specific concerns will trigger a tailored response delivered to patient and/or partner by email.
3	Beginning to recover sexual intimacy as a couple	Recognition that sexual changes will require adaptation by the man and the partner, likely use of sexual aids and a need to adopt a flexible approach to sexual interactions.	Video: A sex therapist encourages the work on sexual recovery, movement towards a ‘new sexual normal.’
			Education: Coping with sexual changes, role of grief, management of expectations, understanding the role of the partner, flexible approach to sexual intimacy, potential barriers, review of sexual aids for men and partners, sensate focus exercises.
			Activity: Draw body maps to use for sensate focus exercises. Staying connected.
			Request for tailored strategies: Specific concerns will trigger a tailored response delivered to patient and/or partner by email.
4	Integrating sex into life’s routine	Acceptance of “new sexual normal.”	Video: Couple talking about engaging in regular, intentional sexual activity.
			Education: Importance of communication, finding time to have sexual activity, review of choices of sexual aids, role of non-penetrative sex and affection, mutuality in the sexual relationship.
			Activity: Develop a plan for regular sexual activity, including sexual aids and how each partner will contribute. Identify barriers to regular sexual activity and how they can be overcome. Staying connected.
			Request for tailored strategies: Specific concerns will trigger a tailored response delivered to patient and/or partner by email.
5	Effective sexuality after prostate cancer	Review the work on sexual recovery with a sense of accomplishment	Video: Couple discusses where they are 6 months after treatment.
			Education: Review of sexual changes, rehabilitation, use of sexual aids, expanded sexual repertoire, grief work, communication, participation in the study.
			Activity: Worksheet to identify sexual activities practiced, including frequency, aids used, the degree to which emotional acceptance was reached, and what goals were reached. Staying connected.
			Request for tailored strategies: Specific concerns will trigger a tailored response delivered to patient and/or partner by email.
10	How to speak to medical providers about sexual problems	Empower men and partners to feel comfortable addressing sexual concerns	Video: Couple talking about how difficult it can be to discuss sexual problems with their provider, and how to overcome barriers.
			Education: Emphasize the importance of engaging providers about sexual concerns, discuss strategies for preparing to do it, discuss roles that the man and partner can take, encourage rehearsal.
			Activity: Review of worries, priority list of questions important to patient and partner, role-play.
			Request for tailored strategies: Specific concerns will trigger a tailored response delivered to patient and/or partner by email.

***Additional Modules that will be part of the website, but not part of the Randomized Control Trial***

**Module 6: Maintaining a sexual relationship for the long term**

**Module 7: One month after biochemical relapse**

**Module 8: Sexuality during advanced stages of prostate cancer**

**Module 9: Overcoming barriers to sexual recovery**

The approach to the intervention is mindful of the a) biopsychosocial nature of sexuality; b) need to include information for gay men, c) representation of diverse cultural and ethnic groups, d) recognition that recovery does NOT mean returning to baseline, and d) the need to involve the partner at each stage of the sexual recovery process. The modules are tailored so that the content is relevant and appropriate according to (1) partnership, (2) prostate cancer treatment type and (3) sexual orientation. The language comprehension of the intervention reflects users with a 6th grade education. Although all modules were developed with the requisite tailoring during this project, only the 6 (1,2,3,4,5,10) modules, addressed to couples, will be tested initially in the RCT. 

Each intervention module has the following sections: 1) video introduction featuring an individual or a couple who have gone through the prostate cancer experience or a relevant expert, such as a sex therapist, 2) educational content, 3) an activity for couples to engage in online, and 4) an opportunity to identify patient and partner concerns. Each intervention module lasts approximately 45 min or less. After each module, participants will receive, via email, tailored strategies, based on concerns they raised during the immediately preceding module’s activity.

### Procedure and adherence to treatment

Participants who pass through the screening process will be recruited in clinics or by telephone, depending on their availability. They will be consented during clinic appointments or by mail. Each couple will be given an envelope with their study identification number (ID) and login information with which to enroll into the True NTH website. The login password is coded to randomize the participating couple into the intervention or control arm of the study. After logging in, participants will provide basic demographic information. Patients will be asked to identify the gender of their partner and type of treatment they will undergo. Once this information is entered, the couple will be sent to surveys tailored to their sexual orientation and treatment type. They will then be asked to fill out baseline surveys. The participants who are randomized into to the intervention arm will access content tailored to their treatment and sexual orientation. Participants who do not fill out their baseline surveys will receive up to 3 phone calls from the study coordinator at participating sites. Those who do not fill out baseline surveys will not be able to continue in the study. Participants who do not log into a given module will receive up to 3 email reminders which are programmed into the intervention. Participants will be allowed to move forward in the intervention even if they do not log into the 3-month surveys to enable tracking their interest in the web-based support.

Participants in the intervention arm will receive up to 3 email reminders when their successive modules become available. They will be able to continue in the intervention even if they do not access the modules. Participants in the control arm will access a link to a “Sexual Health after Cancer” web page of the American Cancer Society (ACS). They will receive 3 email reminders. The ACS website link will be available each time these participants access the True NTH website, for example when they fill out their 3 month and 6 month surveys. They can also return to the True NTH website at will throughout the time of the study. As participants will enter the study prior to treatment, their study participation will continue for approximately 7 months.

### Measures

Measurement timeline that includes both the primary measure and secondary measures is outlined in Table 2. Description of the measures is included in this section.

**Table 2:** Study measures and data collection timeline

Variable	Measure	Assessment					
		*Baseline*		*3 Months*		*6 Months*	
		Patient	Partner	Patient	Partner	Patient	Partner
PRE-EXISTING AND CLINICAL CHARACTERISTICS							
Demographics	Demographic survey	X	X			X	X
Comorbidities	Katz Comorbidity Questionnaire Charlson Comorbidities Index	X	X			X	X
Clinical data	Medical Chart Review	X		X		X	
Patient prostate cancer related symptoms	Expanded Prostate Cancer Index Composite (EPIC)	X		X		X	
Patient expectation of sexual function	Expanded Prostate Cancer Index Composite-Exp (EPIC-Exp)	X					
Partner sexual function							
Female	Female Sexual Function Index (FSFI)		X		X		X
Male	The International Index of Erectile Function (IIEF)		X		X		X
Expectations	Expectation of Success of Treatment	X	X				
PRIMARY OUTCOME							
Global Satisfaction with Sex Life	Patient Reported Outcome Measure Information System (PROMIS) Global Sexual Satisfaction	X	X			X	X
SECONDARY OUTCOMES							
Interest in sex	PROMIS Interest in Sex Life	X	X	X	X	X	X
Sexual activity	PROMIS Sexual Activity	X	X	X	X	X	X
Resources							
Use of Aids	PROMIS Use of Aids	X	X	X	X	X	X
Knowledge	Knowledge Building Assessment	X	X	X	X		
Dyadic Coping	Dyadic Coping Inventory (DCI)	X	X			X	X
Grief	Prolonged Grief about the Loss of Sexual Function			X	X	X	X
Quality of Life	Quality of Life EQ-5D	X	X	X	X	X	X
Satisfaction with Intervention	Satisfaction with Intervention					X	X

**Patient Reported Outcome Measure Information System (PROMIS) Global Satisfaction with Sex Life** [49] measures patients’ and partners’ overall satisfaction with their sex life. Participants respond to seven items on a 5-point scale. A higher score reflects greater satisfaction (Cronbach alpha = 0.92).

**PROMIS Interest in Sex Life** [49] measures patients’ and partners’ interest in sex. Participants will respond to four items on a 5- point Likert scale. A higher score will indicate a higher level of interest (Cronbach alpha = 0.87).

**PROMIS Sexual Activity** [49] is a 13-item measure that evaluates the frequency and type of sexual activity. It is a descriptive measure.

**PROMIS Use of Aids** [49] is a 7-item measure that assesses the use of hormones, personal lubrications, medications, or devices intended to allow for or improve sexual function. These items are intended to be “stand alone” items and do not comprise a unidimensional scale.

**Knowledge Building Assessment** is an 18-item measure that was adapted from a previous study. Ten items assess knowledge about the effect of prostatectomy on erections, understanding of penile rehabilitation, erectile aids, expectations for erection recovery, flexible couple sexuality, and barriers to sexual recovery. The items are categorical variables and will be dichotomized. A summary score will be calculated.

**Expanded Prostate Cancer Index Composite-26 (EPIC)** [50] is a 26-item prostate cancer health-related quality of life (HRQOL) instrument that assesses urinary, bowel, sexual, and hormonal treatment-related symptoms of prostate cancer. Participants respond to a Likert scale. A higher score reflects higher function (Cronbach alpha = 0.93). Sexual function cut-off scores of the EPIC are: 0–33 (severe ED), 34–45 (moderate ED), 46–60 (mild/moderate ED), 61–75 (mild ED), and above 75 (no ED).

**EPIC-Exp** [50] is a measure parallel to the EPIC, but focused on expectations of functional recovery 1 year after treatment.

**Female Sexual Function Index (FSFI)** [51] is a 19-item questionnaire measure of sexual functioning in women. It assesses six domains of sexual functioning: sexual desire, arousal, lubrication, orgasm, satisfaction and pain. A total score is a sum of the domain scores. Participants respond to a Likert scale.

**Dyadic Coping Inventory (DCI)** [52] is a 16-item questionnaire that assesses stress communication and dyadic coping as perceived by (1) each partner about their own coping (what I do when I am stressed and what I do when my partner is stressed), (2) each partner’s perception of the other’s coping (what my partner does when he or she is stressed, and what my partner does when I am stressed), and (3) each partner’s view of how they cope as a couple (what we do when we are stressed as a couple). Participants respond on a Likert scale. A higher score reflects higher dyadic coping (Cronbach alpha for subscales = 0.71–0.92)*.*

**Prolonged Grief about the Loss of Sexual Function** [53] is a 22-item scale that was adapted for sexual function loss from the Prigerson’s validated measure of prolonged grief in bereavement. Participants respond to a Likert scale.

**The International Index of Erectile Function (IIEF)** [54] is a 15-item scale with subscales that measure erectile function, orgasmic function, sexual desire, intercourse satisfaction and overall satisfaction. Participants respond on a Likert scale. A higher score reflects higher sexual function. (Cronbach alpha for subscales and overall IIEF is 0.91, 0.92, 0.92, 0.77, 0.73, 0.74, respectively). As the IIEF was validated for heterosexual sex with vaginal intercourse as the expected activity, several additional items will be added for gay participants, but not included in scoring to inform about gay sexual experience.

**Expectations of Success of Treatment** [55] is a 10-item measure, adapted from a mental health measure for confidence regarding treatment for sexual dysfunction. It measures confidence in the treatment and in the outcome of the treatment. Participants respond on a Likert scale. A higher score reflects greater confidence in the success of treatment.

**Quality of Life EQ-5D** [56] is a standardized QOL instrument for use as a measure of health outcomes. It essentially consists of 2 pages – the EQ-5D descriptive system, and the EQ visual analogue scale (EQ VAS). The EQ-5D-3 L descriptive system comprises the following 5 dimensions: mobility, self-care, usual actives, pain/discomfort and anxiety/depression. Each dimension has 3 levels: no problems, some problems, extreme problems.

**Demographic/Lifestyle Data** Demographic data such as age, partnership status, race/ethnicity, household income, education, weight, height and cigarette smoking status will be sought.

**Comorbidities** [57], such as heart disease, arthritis, diabetes and others, will be collected on a self- reported basis from both the patient and partner.

**Satisfaction with Intervention** is a 10-item survey that was developed for this study by the study team and has face validity. Participants respond to a Likert scale. Individual items are summed into a total score. A higher score means higher satisfaction.

**Clinical data** will be abstracted from patients’ health records, including PSA and clinical stage of prostate cancer at diagnosis, Gleason score, comorbidities as rated per the Charlson Comorbidities Index [58], etc. For surgical patients, pathological stage, margin status (positive/negative), pathologic Gleason score and 3-month post-surgery PSA at 3 will be collected.

### Data analysis

#### Endpoints

The primary endpoint for this study will be the PROMIS Sexual Satisfaction measure at the 6-month follow-up, a multi-item validated assessment of satisfaction with sex life that was designed to have a mean of 50 and a standard deviation of 10 in the general population. Secondary endpoints will consist of the other functional and QOL measurements, comorbidities, and satisfaction assessments (Table 2).

### Analysis

The primary endpoint of PROMIS Satisfaction with Sex Life at 6 months is expected to be a continuous measurement that is normally distributed. If not, we will transform the data for this outcome to approximate normality. Since the primary outcome is measured on both patients and partners and randomization was done at the level of each dyad, we will assess the impact of the intervention using the Actor-Partner Interaction Model (APIM) [59]. The APIM is a mixed-effects linear regression model that allows us to simultaneously estimate the association of partner characteristics with partner and patient outcomes, as well as simultaneously estimate the association of patient characteristics with both outcomes. The APIM incorporates random effects to account for the correlation between patient and partner outcomes from the same dyad. Our base model will contain (1) an indicator of intervention versus control, (2) baseline PROMIS Satisfaction with Sex Life, (3) a indicator of partner versus patient, (4) interaction terms of (3) with (1) and (2), (5) a random partner effect to account for variation among partners, (6) a random patient effect to account for variation among patients, and (7) a third random effect to account for the correlation (non-independence) between outcomes measured from the same dyad. From this base model, we can assess not only the relative impact of the intervention among dyads, but also the differential impact of the intervention on partners and partners.

We can then expand our base model to include other partner characteristics and/or patient characteristics to assess whether or not these characteristics moderate or mediate the effect of the intervention for the patient and the partner. For example, we could include baseline satisfaction with sex life of the partner and the patient and see if either or both factors moderate or mediate the effect of the intervention on 6-month satisfaction with sex life. We note that because dyads were randomized to the intervention, we expect dyad level characteristics to have little effect in our model because those factors should be nearly balanced among the two treatment arms. Thus, adjustment for them is unnecessary, except to gain statistical power. The random effects will allow us to estimate the remaining variability of outcomes among partners, among patients, and the correlation of outcomes within each dyad, that is not explained by our APIM.

All secondary endpoints will be analyzed with an APIM exactly as described for the primary endpoint. It is possible that some secondary endpoints, even after transformation, will have slightly skewed distributions. However, random effect models are fairly robust to slight deviations from normality.

Along with outcome data analyses, we will conduct an analysis of the logistics of participant activity. This will include participant engagement with the modules of the intervention on the web, the need for reminders, time spent on each module and number of times each module was accessed.

### Power calculations

Basing power calculation on the average adjusted PROMIS Satisfaction with Sex Life score of 50 (standard deviation = 10) provided in the PROMIS scoring and anticipating Sexual Satisfaction scores for the intervention group vs. control group to be 50 vs. 45 based on expert opinion, a sample size of 128 patients and 128 spouse/partners will provide 80% power at the 5% significance level to detect this difference using a two-tailed t-test. Adding 10%, or 14 dyads (including an extra one to get to an even number to allow the same sample size in each arm), to our total to account for the anticipated dyads that will be lost to follow-up prior to study completion, we will recruit 140 dyads (280 individuals) for this study (expected to be 70 couples on each study arm).

### Ethics

The study will be coordinated by the University of Michigan, the protocol has been approved by the University of Michigan Institutional Review Board (IRB) and by the IRBs of all the other participating sites. The process of the study and protocol modifications, if needed, will be discussed in weekly meetings with all the sites and amendments filed to each institution’s IRB. Data will be collected by the University of Michigan Center for Healthcare Communications Research (CHCR) and stored securely on virtualized servers with a robust back up system. All participants must sign informed consent before enrolling in the study.

## Discussion

Building on the acceptability of a previous web-based intervention for couples after prostate cancer treatment, which was shown to be as effective as a face-to-face intervention [20], our intervention represents several innovations. It is based on an evidence-based theoretical model that incorporates both a biopsychosocial approach to sexuality and the grief process, as a path to recovery. Unlike any previous sexual health intervention, it is interactive and has a personalized approach achieved through tailoring to treatment, partnership and sexual orientation. When fully implemented, it will provide support for men with prostate cancer and their partners along the trajectory of the illness, including during disease progression and in the metastatic stage. This approach recognizes both the hard work that men and couples undertake when they desire to maintain their sexual viability after prostate cancer treatment, and the fact that men and partners benefit from staying close during all phases of illness. [60] Patients’ and partners’ quest to maintain sexual intimacy will be reinforced by modules that provide coaching for discussions of sexual concerns between the patient, partner and healthcare provider and alert them to barriers to sexual recovery with relevant strategies.

The evaluation of the intervention is also innovative. In particular, we are investigating satisfaction with sex life, which may be applicable to a broad array of men, including older men and their partners who may be able to attain/maintain satisfaction with their sex life despite compromised sexual function [61]. Furthermore, the study includes many variables that are critical and relevant to a successful sexual recovery, such as sexual function, grief about the loss of sexual function, dyadic coping, knowledge acquisition, some of which have been primary outcomes in previous studies. Finally, our analytic approach allows us to evaluate the couple which experiences prostate cancer as a unit with reciprocal influence on each other’s outcomes. Our goal is to provide an accessible, comprehensive and individually relevant supportive structure for recovery from this well-recognized long-term treatment effect [62], and to contribute positively to patients’ and partners’ quality of life in survivorship. When the study is completed, we plan to publish the findings. Subsequently, the intervention will be modified, as needed, based on study findings and offered in full on the Movember Foundation-True NTH website to all prostate cancer survivors and their partners.

## Abbreviations

ACS: American Cancer Society
ADT: Androgen deprivation therapy
APIM: Actor-Partner Interaction Model
CCI: Charlson Comorbidities Index
CHCR: Center for Health Communications Research
DCI: Dyadic Coping Inventory
ED: Erectile dysfunction
EPIC: Expanded Prostate Cancer Index Composite
EQ-5D: European Quality of Life – 5 Dimensions
Est.: Estimated
FSFI: Female Sexual Function Index
HRQOL: Health Related Quality of Life
IIEF: International Index of Erectile Function
IRB: Institutional Review Board
PROMIS: Patient Reported Outcome Measure Information System
QOL: Quality of Life
RCT: Randomized Controlled Trial
TRT: Testosterone replacement therapy
VAS: Visual Analog Scale
